# Efficacy of acupuncture for depression: a systematic review and meta-analysis

**DOI:** 10.3389/fnins.2024.1347651

**Published:** 2024-04-30

**Authors:** Yawen Tan, Ruqian Duan, Chuanbiao Wen

**Affiliations:** School of Intelligent Medicine, Chengdu University of Traditional Chinese Medicine, Chengdu, China

**Keywords:** depression, acupuncture treatment, pharmacotherapy, randomized controlled trials, meta-analysis

## Abstract

**Introduction:**

Depression is a pervasive mental health challenge with substantial global ramifications. Contemporary therapeutic strategies predominantly consist of psychological interventions and pharmacological treatments. Acupuncture, deeply rooted in ancient traditions and bolstered by a plethora of clinical trials, demonstrates considerable efficacy in depression. This study aims to elucidate the efficacy and safety of acupuncture as a standalone therapy for depression patients by reviewing randomized controlled trials that compare acupuncture treatment with conventional drug therapy.

**Methods:**

Comprehensive searches were conducted across six databases in both Chinese and English: CNKI, Wanfang, VIP, Embase, Medline, and CENTRAL. The literature search spanned from 1 July 2013, to 1 July 2023. Two researchers independently carried out literature screening and data extraction. Review Manager 5.4 was utilized for data analysis and bias risk assessment. A total of 20 randomized controlled trials were included in the qualitative synthesis, involving 1,376 participants and 43 relevant acupoints.

**Results:**

The Meta-analysis results, based on the HAMD scale scores, revealed that acupuncture regimens (RR: −1.63; 95% CI: −2.49 to −0.76; *P* = 0.0002; *I*^2^ = 86%; *n* = 1,668) were significantly more effective compared to standalone medication. Moreover, efficacy metrics from the HAMD highlighted a substantial advantage of acupuncture (RR: 2.6; 95% CI: 1.6 to 4.23; *P* = 0.0001; *I*^2^ = 0; *n* = 614). Further assessments utilizing SERS and TESS demonstrated a lower incidence of side effects and adverse outcomes in the acupuncture group.

**Conclusions:**

Acupuncture, when compared to conventional pharmacotherapy, exhibits significant efficacy as a standalone treatment after four weeks of intervention, with fewer side effects and adverse reactions. However, further investigation is needed to determine the most effective acupoints and appropriate types of acupuncture techniques for treating depression.

**Systematic review registration:**

https://www.crd.york.ac.uk/prospero/, identifier CRD42023443711.

## 1 Introduction

Depression, a prevalent affective disorder, clinically manifests as psychological impairments, such as profound and sustained mood despondency, anhedonia, and cognitive dispersion. It is also accompanied by somatic manifestations, including anorexia and fatigue (Li et al., 2023). Clinically, it occurs as the complication of diabetes (Boehmer et al., 2023), sleep disorders (Zhang et al., 2022), anxiety (Khoo et al., 2022), and coronary heart disease (Lu et al., 2022). Furthermore, there is a growing trend of depressive manifestations observed following conditions such as strokes (Huang et al., 2022), childbirth (Xu et al., 2022), the perimenopausal period (Yang et al., 2023), and various surgeries (Yu et al., 2023). In recent times, there has been a notable rise in the prevalence of depression, causing significant harm not only to affected individuals but also triggering pronounced socio-economic repercussions on families and society. According to the World Health Organization assessment, the global incidence of depression is approximately 15%, and this figure is on the rise (World Health Organization [WHO], 2018). Projections suggest that by 2030, depression is poised to become the leading economic burden in global health paradigms (Bayes et al., 2020). Currently, the primary treatment methods for depression mostly involve a combination of psychological counseling and drug therapy. The drugs commonly prescribed include selective 5-HT (5-hydroxytryptamine) represented by fluoxetine hydrochloride capsules and reuptake inhibitors (Chen and Yuan, 2021), followed by tricyclic antidepressants like paroxetine, fluoxetine, sertraline, amitriptyline, trazodone, etc (Ma et al., 2023). However, there may be a delay in achieving the desired steady-state plasma concentrations post-administration of these pharmaceutical agents (Zhu et al., 2017). Additionally, post-treatment adverse events are not uncommon, with an estimated 40% of patients experiencing suboptimal therapeutic efficacy and encountering various adverse effects, including dizziness, somnolence, and weight gain (Pfau et al., 2018). Severe manifestations, such as cardiovascular anomalies, gastrointestinal hemorrhages, sexual dysfunction, and metabolic syndromes, have been reported. The significant recurrence rate of these medications further complicates the treatment trajectory (Tian et al., 2020). Collectively, these factors may lead to compromised patient adherence, ultimately diminishing the overall therapeutic benefit. Consequently, there is a growing academic and clinical motivation to explore alternative and complementary therapeutic modalities.

Acupuncture, recognized as an effective intervention for depression (Hu et al., 2011), offers several advantages over conventional pharmacotherapies. It is characterized as a favorable cost-benefit ratio, a well-established safety profile, a diminished risk of adverse reactions, enhanced therapeutic efficacy, and favorable patient acceptability (Chen et al., 2011). Multiple meta-analyses and systematic reviews affirm the efficacy of acupuncture as an intervention for depression. However, a majority of existing studies centers on exploring acupuncture’s impact on depression accompanied by other comorbidities or its combination with pharmacological treatments. Besides, there is an increasing number of studies focusing on complex treatment approaches aimed at improving efficacy, such as combining acupuncture with psychotherapy (Dong et al., 2017), medication (Li et al., 2018), music therapy (Wijeratne et al., 2022), moxibustion (Luo et al., 2022), acupoint injection (Fan, 2019), or tuina therapy (Meng et al., 2021). Manifestly, no research focuses solely on the efficacy of standalone manual acupuncture or electro-acupuncture in treating uncomplicated depression. Furthermore, questions regarding the optimal duration of acupuncture treatment and the underlying mechanism remain unanswered. Besides, discerning whether the effectiveness of these multimodal approaches stems solely from acupuncture or from synergistic effects with other modalities poses a change.

With this context in mind, our study aims exclusively on primary depression, excluding cases with secondary depression or concurrent comorbidities, and to investigate the efficacy of manual acupuncture and electro-acupuncture as standalone interventions for uncomplicated depression. By isolating depression from other conditions and therapeutic modalities, we aim to gain insights into the specific mechanisms underlying acupuncture’s therapeutic benefits in this population. This research not only informs treatment optimization but also contributes to a deeper understanding of acupuncture’s role in managing depression.

## 2 Materials and methods

### 2.1 Study registration

The protocol for this study has been appropriately registered in PROSPERO, the international prospective register of systematic reviews, with the identifier CRD 42023443711. This registration emphasizes the study’s dedication to transparency and methodological rigor, ensuring that the research adheres to predetermined objectives and methodologies.

### 2.2 Retrieval, methods for research appraisal

We conducted a comprehensive literature search across multiple databases, covering publications from July 1, 2013, to July 1, 2023. Our search encompassed three Chinese databases: China National Knowledge Infrastructure (CNKI), Wanfang Data Knowledge Service Platform, and VIP Chinese Journal Service Platform, as well as three English databases: Embase, Medline, and the Cochrane Controlled Register of Trials (CENTRAL). Our search strategy was not restricted by language. In each database’s native language, we employed the terms “depression” and “acupuncture/electroacupuncture” for our search. To ensure maximum efficacy in our search approach, we tailored our search strategy to suit the nuances of each database. To elucidate our methodology, we present the Embase search as an exemplar in Table 1.

**TABLE 1:** Search strategy of Embase.

Number	Search terms
#1	(acupuncture [Title/Abstract]) OR (electroacupuncture [Title/Abstract])
#2	(depression [Title/Abstract]) OR (major depression [Title/Abstract]) OR (main depression disorder [Title/Abstract])
#3	#1 AND #2

Initially, the therapeutic modalities of acupuncture and electroacupuncture were identified. Subsequently, the research focused on varying intensities of mild depression. The search terms “depression,” “major depression,” and “main depression disorder” were selected. Literature featuring these terms in their titles and abstracts was explored in Embase using the strategy outlined in Table 1. This approach facilitated the preliminary identification of relevant studies within the database and detailed the specific quantity of articles retrieved from each of the six databases. Additionally, it described the volume of literature subsequently screened based on inclusion-exclusion criteria, along with the rationales for their exclusion, as delineated in the results section under study selection.

### 2.3 Inclusion and exclusion criteria

Inclusion Criteria for Qualitative Synthesis of randomized controlled trials (RCTs): (i) Intervention Type: The clinical intervention should exclusively involve acupuncture techniques, including manual acupuncture, scalp acupuncture, auricular acupuncture, intradermal acupuncture, or electroacupuncture. Moxibustion-related interventions are not to be included. (ii) Control Group: Only pharmacological treatments (excluding traditional Chinese medicine and proprietary Chinese medicine) such as paroxetine, fluoxetine, and sertraline are permitted in the control group. (iii) Participant Distribution: The participant ratio between the intervention and control groups should be 1:1, with no statistically significant differences in gender distribution.

To meet the inclusion criteria, studies were required to employ either simple manual acupuncture or electroacupuncture exclusively. Electroacupuncture, an advanced form of manual acupuncture, provides a more potent stimulation to acupuncture points (Han et al., 2021). Scalp and auricular acupuncture, while specifying the needle application sites, are essentially categorized under hand acupuncture. Moxibustion was excluded to avoid the potential effects of heat (i.e., temperature) on treatment efficacy. The control group received Western medications commonly prescribed for depression treatment. Stringent control was maintained over the size and gender ratio of participants in both the intervention and control groups. This rigorous selection aimed to exclude studies lacing in insufficient rigor, such as those with disparities in group sizes or attrition during the experiment, and to minimize potential biases stemming from uneven gender distribution.

Exclusion Criteria: (i) Clinical trials with fewer than 20 participants in either the intervention or the control group. (ii) Studies employing non-acupuncture techniques or combined methods in the intervention group, such as a combination of acupuncture with medication, massage, or moxibustion. (iii) Trials incorporating psychological counseling as an auxiliary treatment modality.

Trials with fewer than 20 participants were excluded due to concerns that clinical trials of such small sample sizes could result in broader confidence intervals and increased inaccuracy. Our study focused on evaluating the comparative efficacy of hand acupuncture as a standalone treatment versus medication alone. Therefore, we excluded trials where the intervention group received a combination of treatments such as acupuncture with medication, acupuncture paired with tuina, or acupuncture alongside psychotherapy.

### 2.4 Research selection and data extraction

The methodology for literature selection proceeded as follows: Two independent researchers conducted systematic literature searches, eliminating redundant publications. Subsequently, the titles and abstracts of the compiled articles were screened independently, followed by a comprehensive review of the full-text articles. Utilizing a standardized data extraction template, relevant information was collected, culminating in a consensus. Upon thorough examination of the full texts, studies meeting inclusion criteria were identified, with RCTs integrated into a qualitative analytical process. Specifically, RCTs covering data from weeks 1 through 6 were included in a quantitative meta-analytic framework. The collected data included authorial credentials, year of publication, sample size, diagnostic criteria, mean age, gender distribution of participants, intervention versus control approach, key pivotal outcomes, and overall research conclusions.

### 2.5 Outcomes

The primary outcomes utilized in this study include the severity of depression assessed by the Hamilton depression rating scale (HAMD), and the therapeutic effectiveness of depression treatment, assessed through the percentage reduction in HAMD scores. The HAMD, devised by Hamilton in 1960, stands as one of the most frequently employed scales in clinical practice for reassuring the severity of depressive symptoms. It is available in three versions: 17-item, 21-item, and 24-item, with the 17-item version being the predominant choice in clinical settings (Zhang, 1998). Assessment of therapeutic efficacy based on the HAMD involves calculating the percentage reduction, calculated as (baseline score–post-treatment score) ÷ (baseline score) × 100%. Remission is defined as a HAMD reduction rate of ≥ 75%; marked effectiveness as a HAMD reduction rate of ≥ 50% but < 75%; moderate effectiveness as a HAMD reduction rate of ≥ 25% but < 50%; and inefficacy as a HAMD reduction rate of < 25% (Shen, 1994).

### 2.6 Statistical, analysis

All statistical analyses were conducted using Review Manager version 5.4. This study employed a random-effects model for the meta-analysis. Dichotomous data, such as the HAMD scores between the acupuncture group and the medication treatment group, and the efficacy rates derived from HAMD reduction percentages, were expressed in terms of risk ratios. For continuous outcomes, the standardized mean difference was calculated with a 95% confidence interval (CI). Longitudinal analysis methods were used to assess the clinical and methodological heterogeneity of the included studies. *I*^2^ tests were applied to analyze statistical heterogeneity among subgroups, with an *I*^2^ value exceeding 50% considered significant. Despite low heterogeneity detection, the random-effects model was applied, recognizing the limitations of heterogeneity tests, especially with a small number of component studies. A difference (*P* < 0.05) was considered to be statistically significant.

## 3 Results

### 3.1 Study selection

**FIGURE 1:**
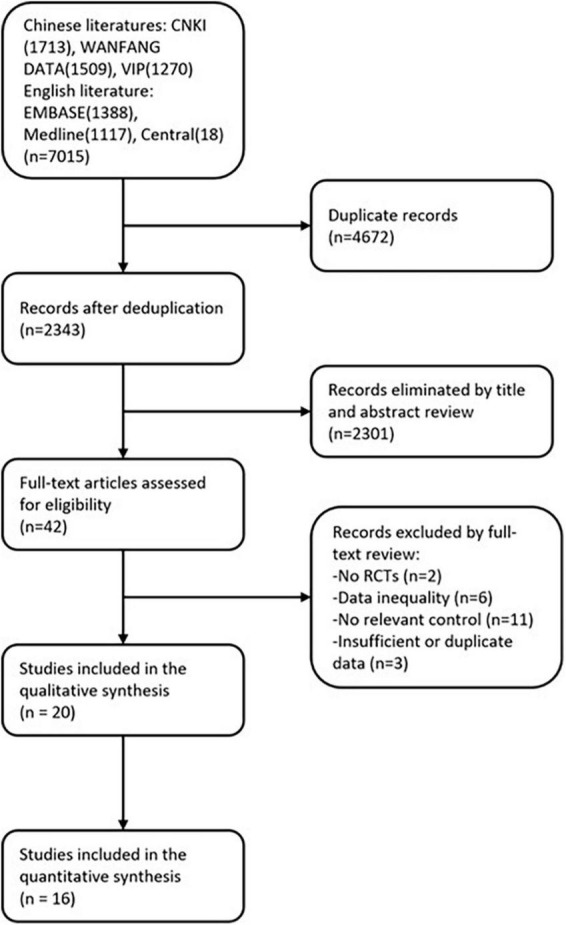
Flow chart of the review process.

As illustrated in Figure 1, our search initially identified a total of 7,015 publications. Following the removal of duplicates (*n* = 3,109), this number was reduced to 42 upon screening of titles and abstracts. Subsequently, after a thorough review of the full texts of these publications, 20 articles met our inclusion criteria. Among the 42 articles initially identified, 22 were excluded for various reasons: 2 were not randomized controlled trials, 11 lacked appropriate controls, 3 had insufficient or duplicated data reporting, and 6 had experimental groups that didn’t match the control groups in terms of participants, resulting in an actual ratio other than 1:1 (including post-dropouts). Consequently, a total of 20 RCTs were ultimately incorporated into the qualitative synthesis process. Among these, 16 studies had data available at weeks 1, 2, 3, 4, 5, and 6, and were subsequently included in the quantitative synthesis process.

### 3.2 Study characteristics

The identified studies, conducted between 2013 and 2023, involved a total of 1376 participants, as outlined in Table 2. These studies were published in both Chinese and English. Diagnostic criteria for depression combined perspectives from both traditional Chinese medicine (TCM) and Western medicine. Western diagnostic criteria were sourced from the International Classification of Diseases, Tenth Edition (ICD-10) for depressive disorders, the Diagnostic and Statistical Manual of Mental Disorders, Fourth Edition (DSM-IV), the Chinese Guideline for the Prevention and Treatment of Mental Disorders (CGPTD), and the Chinese Classification of Mental Disorders, Third Edition (CCMD-3). Traditional Chinese Medicine diagnostic standards were derived from the Clinical Terminology of Traditional Chinese Medicine Syndrome Part (CT TCMDT-S), the National Standard for TCM Disease and Syndrome Diagnosis and Therapeutic Effect (TCM DTES), the Fifth Edition of Traditional Chinese Medicine Internal Medicine (TCM IM-V), and the Principles of the Clinical Research Guide for New Traditional Chinese Medicines (PCG TCM). The HAMD scoring was simultaneously employed as an inclusion criterion.

**TABLE 2:** Summary of randomized controlled trials of acupuncture for patients with depression.

			Participants					
**References**	**Sample size**	**Diagnostic criteria (WM/TCM)**	**Average age (years) IG/CG**	**Sex (M/F)**	**Intervention group regimen**	**Control group regimen**	**Main Outcomes**	**Results**
Li et al., 2019	60	ICD-10; CTTCMDT -S	IG: 34.73 ± 12.70 CG: 36.93 ± 15.31	IG: 10/20 CG: 8/22	AT(*n* = 30) IG: 4 days per week for 8 weeks, 30 min	PT(*n* = 30) CG: sertraline, 50 mg a day for 8 weeks	HAMD	Significant differences in HAMD scores (*p* < 0.05)
Liang et al., 2021	56	ICD-10	IG: 67.51 ± 5.82 CG: 67.31 ± 5.42	IG: 15/13 CG: 14/14	EA(*n* = 28) IG: 5 days per week for 8 weeks, 30 min	PT(*n* = 30) CG: fluoxetine, 20 mg a day for 8 weeks	HAMD WCST MMSE	Significant differences in HAMD scores (*p* < 0.05) Significant differences in WCST scores (*p* < 0.05) Significant differences in MMSE scores (*p* < 0.05)
Ma, 2013	100	DSM-IV; ICD-10	n.r.	IG25/25 CG: 25/25	EA(*n* = 50) IG: 6 days per week for 4 weeks, 30 min	PT(*n* = 50) CG: fluoxetine, 20 mg a day for 4 weeks	HRSD SDS	Significant differences in HRSD scores (*p* < 0.05) Significant differences in SDS scores (*p* < 0.05)
Gao and Tan, 2018	60	n.r.	n.r.	n.r.	EA(*n* = 30) IG: 6 days per week for 4 weeks, 30 min	PT(*n* = 30) CG: fluoxetine, 20 mg a day for 4 weeks	HAMD	Significant differences in HAMD scores (*p* < 0.05)
Xu et al., 2015	60 (52)	ICD-10/ TCM DTES	IG: 40.54 ± 5.42 CG: 41.47 ± 4.07	IG: 14/16 CG: 16/14	AT(*n* = 26) IG: 7 days per week for 4 weeks, 30–40 min	PT(*n* = 26) CG: paroxetine, 20 mg a day for 4 weeks	HAMD TESS	No Significant difference in HAMD scores(*p* > 0.05) Significant difference in TESS scores (*p* < 0.05)
Shi et al., 2013	76	n.r.	IG: 57 ± 9 CG: 55 ± 8	IG: 16/22 CG: 14/24	EA(*n* = 38) IG: 4 days per week for 4 weeks, 30 min	PT(*n* = 38) CG: fluoxetine, 20 mg a day for 4 weeks	HAMD PSQI	Significant differences in HAMD scores (*p* < 0.01) Significant differences in PSQI scores (*p* < 0.01)
Dong and Xu, 2013	60 (58)	CCMD-III/TCM IM; TCM DTES	IG: 42.78 ± 10.39 CG: 41.54 ± 9.53	IG: 7/23 CG: 6/24	AT(*n* = 29) IG: 1 times a day for 30 days, 30 min	PT(*n* = 29) CG: fluoxetine, 20 mg a day for 30 days	HAMD SDS	Significant differences in HAMD scores (*p* < 0.05) Significant differences in SDS scores (*p* < 0.01)
Lv and Gan, 2018	60	CGPTD/TC MDTES	IG: 39.23 ± 9.30 CG: 38.97 ± 10.52	IG: 12/18 CG: 13/17	AT (*n* = 30) IG: 5 days per week for 6 weeks, 30 min	PT(*n* = 30) CG: Fluoxetine, 20 mg a day for 6 weeks	HAMD SERS	No significant difference in HAMD scores(*p* > 0.05) No significant difference in SERS scores (*p* > 0.05)
Song et al., 2013a	80	CCMD — III/TCM DTES	IG: 41.52 ± 12.53 CG: 41.57 ± 12.62	IG: 14/26 CG: 15/25	AT(*n* = 40) IG: 6 days per week for 6 weeks, 25 min	PT(*n* = 40) CG: fluoxetine, 20 mg a day for 6 weeks	HAMD	Significant differences in HAMD scores (*p* < 0.01)
Song et al., 2013b	60	CCMD- III/TCM DTPS	IG: 42.32 ± 12.47 CG: 43.74 ± 12.52	IG: 12/18 CG: 14/16	AT(*n* = 30) IG: 6 days per week for 6 weeks, 25 min	PT(*n* = 30) CG: fluoxetine, 20 mg a day for 6 weeks	HAMD Asberg	No significant difference in HAMD scores(*p* > 0.05) Significant differences in Asberg scores (*p* > 0.05)
Gu et al., 2015	60	CCMD-III/TCM DTES; PCG TCM	IG: 61.83 ± 10.33 CG: 63.53 ± 10.11	IG: 19/11 CG: 18/12	AT(*n* = 30) IG: 6 days per week for 5 weeks, 30 min	PT(*n* = 30) CG: fluoxetine, 20 mg a day for 30 days	HAMD	Significant differences in HAMD scores (*p* < 0.05)
Zhang, 2016	120	DSM-V	n.r.	n.r.	AT(*n* = 60) IG: 7 days per week for 12 weeks	PT(*n* = 60) CG: Citalopram, 20–60 mg a day for 12 weeks	HAMD CGT	Significant differences in HAMD scores (*p* < 0.05) Significant differences in C GT scores (*p* < 0.05)
Chen et al., 2020	60	CCMD-III/TCMIM	IG: 32.1 ± 10.6 CG: 32.3 ± 10.9	IG: 14/16 CG: 13/17	AT(*n* = 30) IG: 5 days per week for 12 weeks, 30 min	PT(*n* = 30) CG: Mirtazapine, 30 mg a day for 12 weeks	HAMD HAMD	Significant differences in HAMD scores (*p* < 0.05) No significant difference in HAMD scores(*p* > 0.05)
Yang and Zhang, 2015	60	ICD-10	IG: 39.73 ± 13.4 CG: 38.43 ± 12.2	IG: 14/16 CG: 13/17	AT(*n* = 32) IG: 5 days per week for 8 weeks, 30 min	PT(*n* = 32) CG: Citalopram, 10–40 mg a day for 8 weeks	HAMD	Significant differences in HAMD scores (*p* < 0.05)
Wang et al., 2016	64	DSM-IV/TCM DTES	IG: 41.3 ± 5.2/ CG: 42.1 ± 4.7	IG: 15/17 CG: 14/18	AT(*n* = 32) IG: 6 days per week for 8 weeks, 30 min	PT(*n* = 32) CG: Mirtazapine, 15–45 mg a day for 8 weeks	SERS	Significant differences in SERS scores (*p* < 0.05)
Wang and Shui, 2015	60	CCMD-III/ DSM-IV	44.2	22/38	AT(*n* = 30) IG: 7 days per week for 6 weeks, 20 min	PT(*n* = 30) CG: Mirtazapine, 15–45mg a day for 6 weeks	HAMD	No significant difference in HAMD scores(*p* > 0.05)
Chen, 2014	52	CCMD-III	44.7	18/34	AT(*n* = 26) IG: 5 days per week for 8 weeks	PT(*n* = 26) CG: fluoxetine, 20 mg a day for 8 weeks	HAMD	No significant difference inHAMD scores(*p* > 0.05)
Zhao, 2015	90	CCMD-III	IG: 43.19 ± 11.37 CG: 42.85 ± 11.69	IG: 21/24 CG: 20/25	AT(*n* = 45) IG: 1 times a day for 90 days, 30 min	PT(*n* = 50) CG: fluoxetine, 20 mg a day for 90 days	HAMD	Significant differences in HAMD scores (*p* < 0.05)
Guo and Miao, 2018	100	n.r.	IG: 45.23 ± 4.95 CG: 44.22 ± 4.97	IG: 27/23 CG: 26/24	AT(*n* = 50) IG: 7 days per week for 5 weeks, 30 min	PT(*n* = 50) CG: fluoxetine, 20 mg a day for 5 weeks	HAMD TESS	No significant difference in HAMD scores(*p* > 0.05) Significant differences in TESS scores (*p* > 0.05)
Wang et al., 2013	60 (48)	DSM-IV	IG: 48.01 ± 13.04 CG: 47.01 ± 10.06	IG: 7/17 CG: 8/16	AT(*n* = 24) IG: 3 times per week for 24 weeks, 20 min	PT(*n* = 24) CG: fluoxetine, 10–60 mg a day for 24 weeks	HAMD	Significant differences in HAMD scores (*p* < 0.05)

M, Western medicine/traditional Chinese medicine; IG/CG, intervention group regimen/control group regimen, M/F, Man/Female; ICD-10, International Classification of Disuses Ten Edition; CTTCMDT-S, clinic terminology of traditional Chinese medical diagnosis and treatment—syndromes; TCM DTES, TCM diagnosis and therapeutic effect standard; AT, acupuncture treatment; PT, pharmacotherapy; HAMD/HRSD, Hamilton depression scale; DSM-IV, Diagnostic and Statistical Manual of Mental Disorders (DSM-IV); EA, electro-acupuncture; WCST, Wisconsin card sorting test; MMSE, mini-mental state examination; SDS, self-rating depression scale; PSQI, Pittsburgh sleep quality index; CCMD-III, classification and diagnostic criteria for mental, disorders in China Third Edition; TCM IM, traditional Chinese internal medicine; CGPTD, Chinese Guidelines for the Prevention and Treatment of Depression; Asberg/SERS, Asberg side-effect rating scale for antidepressant; PCG TCM, Principles of clinical research guidelines for new traditional Chinese medicine; CGT, Clinical global impression, TESS, treatment emergent symptom scale.

Treatment durations varied from 4 weeks (Ma, 2013; Shi et al., 2013; Xu et al., 2015; Li et al., 2019) to 24 weeks (Wang et al., 2013). The interventions in the included studies were confined to acupuncture (such as manual, scalp, or electro-acupuncture), while control interventions primarily consisted of pharmacotherapy (e.g., paroxetine, fluoxetine, or sertraline). Studies lacking HAMD scores or those lasting over 6 weeks were exclusively considered for qualitative synthesis, rather than quantitative, due to their significant heterogeneity compared to those utilizing HAMD scoring within 6 weeks. The results of studies incorporated into quantitative synthesis are outlined in Table 2. Within the qualitative synthesis, all 20 studies compared acupuncture with drug therapy. Seven studies analyzed both HAMD scores and adverse reactions; among them, all 20 employed HAMD, while three also used both HAMD and the Self-rating depression scale (SDS). Of the two (Dong and Xu, 2013; Ma, 2013) studies utilizing both HAMD and SDS, are demonstrated significant differences. Among the 11 studies using only HAMD, seven exhibited significant inter-group differences.

### 3.3 Methodological quality

Acupuncture for depression showed that while most studies demonstrated satisfactory performance in terms of randomized sequence generation and allocation concealment, implementing participant and staff blinding proved challenging due to the inherent nature of the treatment. This resulted in a high risk of bias, whereas the risk of bias concerning blinding for outcome assessment, data completeness, and selectivity of reporting was low.

**FIGURE 2:**
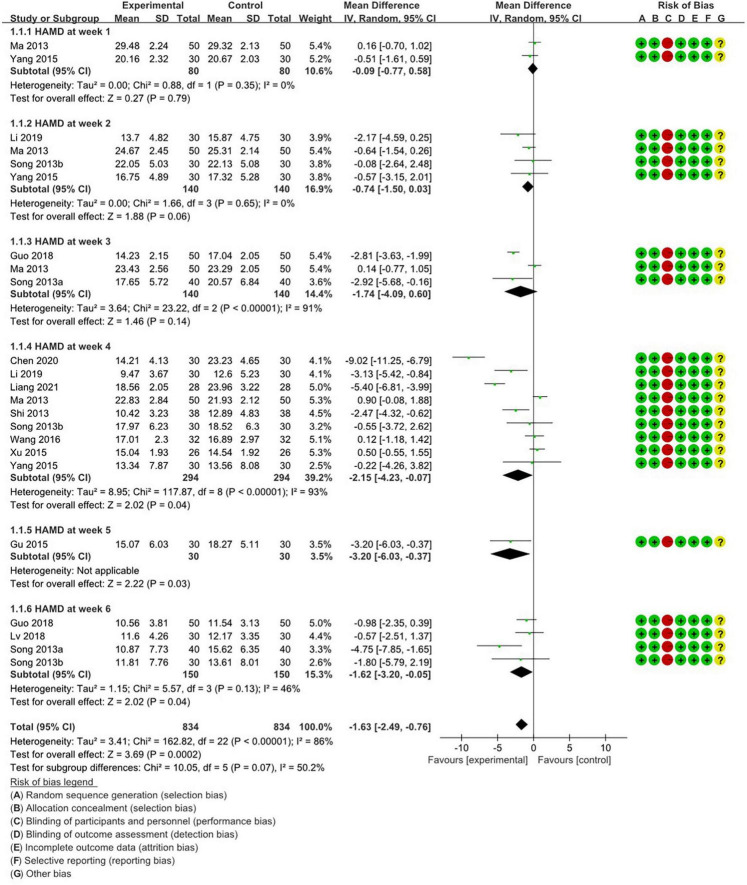
Weekly meta-analysis and bias assessment of acupuncture treatment for patients with depression patients according to HAMD.

Figure 2 summarizes the methodological quality concerning HAMD scores in weekly studies involving depression patients, as derived from the 13 included papers in the final analysis. The results of the Cochrane risk of bias (ROB) analysis were largely consistent. Regarding random sequence generation and allocation concealment, 11 studies showed low ROB. However, for blinding of participants and personnel, all studies exhibited high ROB, as blinding is unfeasible in acupuncture treatment. All studies showed low ROB concerning blinding of outcome assessment, incomplete outcome data, and selective reporting. As for other biases, all studies were deemed unclear.

**FIGURE 3:**
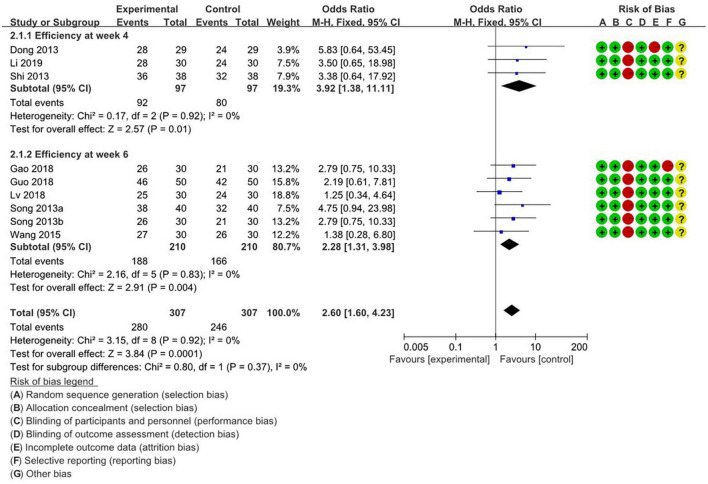
Weekly meta-analysis and bias assessment of acupuncture and moxibustion treatment for depression patients based on the score reduction rate calculated by HAMD.

Figure 3 illustrates the methodological quality of the studies assessing the effectiveness of acupuncture for depression, as measured by HAMD in the final analysis. The results of the Cochrane ROB analysis were generally consistent. Concerning randomized sequence generation and allocation concealment, nine studies had lower ROBs. However, regarding the blinding of participants and staff, all studies had high ROBs, reflecting the inherent limitations of blinding in acupuncture treatment, as discussed above. One study exhibited high ROB in attrition bias, failing to report specific details on dropouts, while another had high ROB in reporting bias, offering only the overall efficacy rate without specific data on significant events. All other studies demonstrated low ROB. Regarding other biases, all studies were categorized as unclear.

**FIGURE 4:**
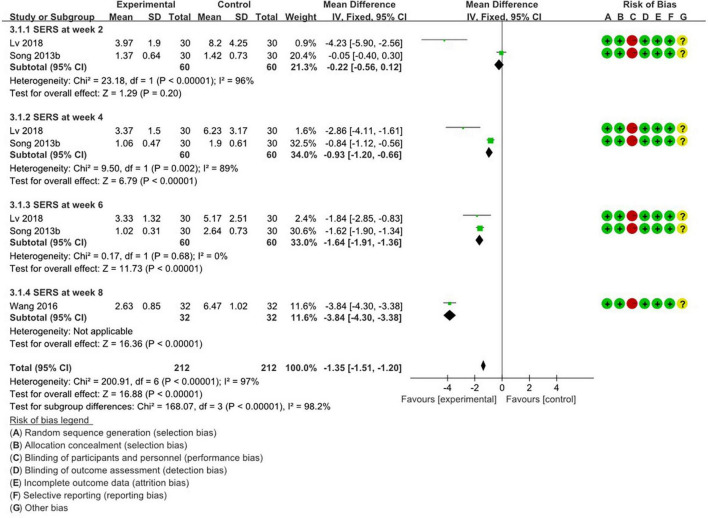
Weekly meta-analysis and bias assessment of acupuncture treatment for depression patients by SERS.

**FIGURE 5:**
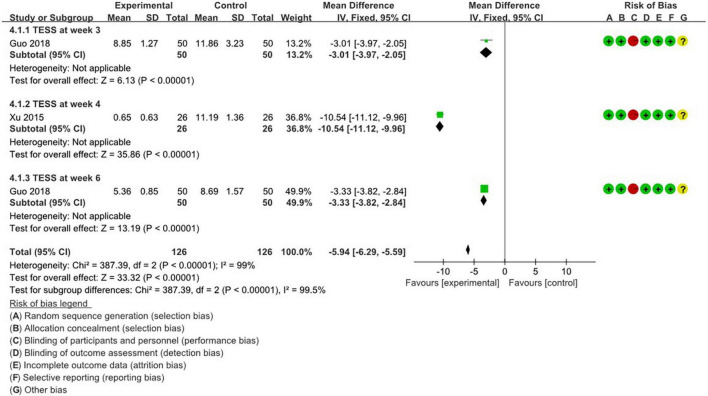
Weekly meta-analysis and bias assessment of acupuncture treatment for depression patients by TESS.

Figures 4, 5 summarize the methodology of the 5 studies incorporated into the final analysis that featured the self-rating depression scale (SDS), self-rating anxiety scale (SERS), and treatment emergent symptom scale (TESS).

Cochrane ROB analysis results were relatively consistent across these studies. Regarding random sequence generation and allocation concealment, all 5 studies had low ROB. However, concerning blinding of participants and personnel, every study showed a high ROB, attributable to the nature of acupuncture treatment. Nonetheless, all studies indicated low ROB in the blinding of outcome assessment, incomplete outcome data, and selective reporting. For other biases, all studies were categorized as unclear.

### 3.4 Meta-analysis

#### 3.4.1 Comparison of time-stratified efficacy of HAMD scores

An analysis of the HAMD scores across various therapeutic weeks divided the treatment groups into two categories. Thirteen studies were stratified into time intervals spanning weeks 1, 2, 3, and 4. The comprehensive meta-analysis (Figure 2) substantiated the notable efficacy of acupuncture in treating depressive disorders (RR: −1.63; 95% CI: −2.49 to −0.76; *P* = 0.0002; *I*^2^ = 86%; *n* = 1668). During the first week, two studies (Ma, 2013; Yang and Zhang, 2015) reported a non-significant impact of acupuncture (RR:−0.09; 95%; CI: −0.77 to 0.58; *P* = 0.79; *I*^2^ =; *n* = 160). This sentiment was echoed in the subsequent week by four studies (Ma, 2013; Song et al., 2013b; Yang and Zhang, 2015; Li et al., 2019) the sentiment was echoed (RR: −0.74; 95%; CI; −1.5 to 0.03; *P* = 0.06; *I*^2^ = 0; *n* = 280). In the third week, three studies (Ma, 2013; Song et al., 2013a; Guo and Miao, 2018) reiterated this insignificance (RR: −1.74; 95%; CI: −4.09 to 0.6; *P* = 0.14; *I*^2^ = 91%; *n* = 280). However, the fourth week witnessed nine studies (Ma, 2013; Shi et al., 2013; Song et al., 2013b; Xu et al., 2015; Yang and Zhang, 2015; Wang et al., 2016; Li et al., 2019; Chen et al., 2020; Liang et al., 2021) championing acupuncture’s substantial effect. By week 5, one study (Gu et al., 2015) reported a significant effect (RR = −3.2; 95%; CI: −6.03 to −0.37; *P* = 0.03; *n* = 60), and by week 6, four studies (Song et al., 2013a,b; Guo and Miao, 2018; Lv and Gan, 2018) observed a significant therapeutic effect (RR = −1.62; 95%; CI: −3.2 to −0.05; *P* = 0.04; *I*^2^ = 46%; *n* = 300).

#### 3.4.2 Comparison of time-stratified efficacy of HAMD-derived efficacy rates

Meta-analysis comparing acupuncture with drug treatment was conducted, grouping nine studies based on their treatment durations of 4 and 6 weeks. Overall, the meta-analysis indicated a significant therapeutic effect of acupuncture for depression (RR: 2.6; 95%; CI: 1.6 to 4.23; *P* = 0.0001; *I*^2^ = 0; *n* = 614). At the 4-week mark, three studies (Dong and Xu, 2013; Shi et al., 2013; Li et al., 2019) reported a significant therapeutic effect (RR: 3.92; 95%; CI: 1.38 to 11.11; *P* = 0.01; *I*^2^ = 0; *n* = 194). Conversely, at the 6-week mark, six studies (Song et al., 2013a,b; Wang and Shui, 2015; Gao and Tan, 2018; Guo and Miao, 2018; Lv and Gan, 2018) identified a notable therapeutic effect (RR: 2.28; 95%; CI: 1.31 to 3.98; *P* = 0.004; *I*^2^ = 0; *n* = 420).

#### 3.4.3 Comparison of time-stratified efficacy of SERS scores

Analyzing the SERS scores across various treatment intervals, the meta-analysis assessed the side-effect profiles of both acupuncture and drug regimens. Holistically, acupuncture manifested a diminished susceptibility to antidepressant-related side effects (RR: −1.51; 95%; CI: −1.51 to −1.2; *P* < 0.00001; *I*^2^ = 97%; *n* = 424). Three studies were examined over treatment periods of 2, 4, 6, and 8 weeks. By the 2-week mark, two studies (Song et al., 2013b; Lv and Gan, 2018) did not find any notable advantage in terms of side effects (RR: −0.22; 95%; CI: −0.56 to 0.12; *P* = 0.2; *I*^2^ = 96%; *n* = 120). By the third week, two studies (Song et al., 2013b; Lv and Gan, 2018) revealed that acupuncture had a significant effect on patients with depression (RR: −0.93; 95%; CI: −1.2 to −0.66; *P* < 0.00001; *I*^2^ = 89%; *n* = 120). At the 6-week mark, similar findings were observed in these studies (Song et al., 2013b; Lv and Gan, 2018), showing clear benefits (RR: −1.64; 95%; CI: −1.91 to −1.36; *P* < 0.00001; *I*^2^ = 0; *n* = 120). Finally, at 8 weeks, one study (Wang et al., 2016) highlighted a pronounced advantage (RR: −3.84; 95%; CI: −4.3 to −3.38; *P* < 0.00001; *n* = 120).

#### 3.4.4 Comparison of time-stratified efficacy of TESS scores

Meta-analysis was conducted to compare the adverse effects of acupuncture with drug treatment. Generally, the meta-analysis indicates that acupuncture exhibits a significant advantage over drug treatment in TESS scores (RR: −5.94; 95%; CI: −6.29 to −5.59; *P* < 0.00001; *I*^2^ = 99%; *n* = 252). Two studies were grouped based on 3, 4, and 6-week treatment durations. At 3 weeks, one study (Guo and Miao, 2018) found a notable advantage in adverse effect scores with acupuncture (RR: −3.01; 95%; CI: −3.97 to −2.05; *P* < 0.00001; *n* = 100). At the 4-week treatment mark, another study (Xu et al., 2015) reported that acupuncture had a distinct advantage in terms of adverse reaction scores for treating depression (RR: −10.54; 95% CI: −11.12 to −9.96; *P* < 0.00001; *n* = 52). Similarly, at week 6, the same study (Guo and Miao, 2018) reached the same conclusion (RR: −3.33; 95%; CI: −3.82 to −2.84; *P* < 0.00001; *n* = 100).

## 3.5 Acupuncture points

As shown in Table 3, among the 19 studies reviewed, only one (Zhang, 2016) did not specify the acupuncture points used. Collectively, these research papers incorporated 43 distinct acupuncture points. Each acupoint was selected in various combinations, with frequencies ranging from 1 and 14 instances. Frequency analysis revealed that the DU 20 point was the most frequently chosen, appearing in over 70% of the studies. Following closely, EX-HN 03, LR 03, and PC 06 were prevalent, appearing in 40–50% of the investigations. SP 06 and HT 07 were recurrent, found in 30–40% of the studies, while ST 36 and DU 24 were utilized in 25–30% of them. Points EX-HN 01 and GB 20 were chosen in 20–25% of the studies. Furthermore, a variety of other acupoints, including RN 12, KI 03, RN 17, EX-HN 05, RN 05, temporal three-needle, and BG 13, were featured in 10–20% of the studies. Additionally, several other points were strategically chosen based on symptomatology and included but were not limited to, KI 06, KI 01, SJ 17, GB 34, and DU 14. These acupoints appeared in less than 10% of the studies.

**TABLE 3:** Acupuncture points selected for the treatment of depression.

	Frequency of use	Acupuncture points
Acupuncture	> 70% (most commonly used)	DU 20
	40–70% (commonly used)	EX-HN 3; LR 3; PC 6
	20–40% (sometimes used)	HT 7; SP 6;ST 36; DU 24; EX-HN 1; GB 20;
	10–20% (occasionally used)	RN 12; KI 3; RN 17; EX-HN 5; BL 24; Temporal., Three-Needle; BG 13; LI 4
	< 10% (rarely used)	KI 6; KI 1; SJ 17; GB 34; GB 14; SP 10; BL 18; BL 15; BL 20; BL 13; BL 23; ST 25; GB 15; ST 25; DU 26; GB 7; DU 21; GB 19; DU 17; GB 8; RN 04; BL 17; DU 16; ST 40; DU 14; PC 7; EX-HN 18; Sisanzhen

In the comprehensive assessment of the 20 studies, the number of acupuncture points allocated for each depression patient varied from 2 to 15. Given the heterogeneity in clinical manifestations during depressive episodes, core acupuncture points were often supplemented by additional, customized selections. For syndrome differentiation: Liver qi stagnation often involved a combination of BL 18, LR 14, LI 04, LR 03, and RN 17. Qi transforming into fire led to selections such as LR 02, ST 44, SJ 06, and GB 43. Disturbances marked by melancholic spirit injury typically incorporated PC 06, HT 05, and BL 15. Instances of heart and spleen deficiency favored BL 15, BL 20, and ST 36. For yang deficiency, additional points RN 05 and RN 04 were recommended. Yin deficiency accompanied by hyperactive fire frequently included selections such as BL 23, KI 03, SP 06, and PC 06 (Song et al., 2013a,b, Wang et al., 2013; Xu et al., 2015). Symptomatic Adjustments: For anxiety and irritability, LI 04 and PC 06 were added. Fatigue and weakness warranted RN 04, ST 36, and SP 06. Insomnia required KI 06, BL 62, and Anmian acupoints, while sleep disorders necessitated HT07, and SiShencong. dizziness called for GB 20, LR 03, whereas headache were addressed with ST 08, GB 08; with palpitations and chest tightness take PC 06, RN 07; dystocia take SJ 05, LR 14, GB 34. Stomach pain was addressed with PC 06, RN 12, and ST 36. Loss of appetite required the addition of SP 03 and SP 04. Abdominal distension necessitated ST 25 and ST 37. Constipation was treated with SJ 06 and ST 25 (Song et al., 2013a,b; Yang and Zhang, 2015). Although the selection of specific allotment points varied slightly among different studies, they all adhered to the principles of acupuncture diagnosis and treatment.

## 4 Discussion

### 4.1 Efficacy and safety of acupuncture for depression

In an extensive review of 7,015 articles, only 20 studies met the stringent criteria for inclusion and exclusion, involving a total of 1,376 patients diagnosed with depression. Among these, 16 studies were selected for comprehensive quantitative meta-analysis, with no limitations on the duration or severity of the illness. The primary outcomes assessed were the scores on the HAMD and the efficiency ratings derived from HAMD, while secondary outcomes included assessments from the SDS, SERS, and TESS. The meta-analysis demonstrated that, across the 16 randomized controlled trials, participants undergoing acupuncture exhibited significant improvements in symptoms after just four weeks of treatment compared to those receiving conventional medication, along with experiencing fewer side effects and adverse reactions. Given the global recognition of HAMD as a tool for assessing depression severity and the widespread clinical use of SDS, SERS, and TESS, the observed improvements in these scores provide compelling evidence of acupuncture’s potential efficacy in treating depression.

A pooled meta-analysis of 11 studies using HAMD scores as a measure confirmed the significant efficacy of acupuncture for depression (RR: −1.63; 95% CI: −2.49 to −0.76; *P* = 0.0002; *I*^2^ = 86%; *n* = 1,668). Nine meta-analyses using HAMD-derived efficacy rates as an instrument showed that acupuncture exhibited significant efficacy in depression (RR: 2.6; 95%; CI: 1.6 to 4.23; *P* = 0.0001; *I*^2^ = 0; *n* = 614). Three studies showed that acupuncture reduced susceptibility to antidepressant-related side effects (RR: −1.51; 95%; CI: −1.51 to −1.2; *P* < 0.00001; *I*^2^ = 97%; *n* = 424). In a meta-analysis involving 2 TESS score studies, acupuncture had a significant advantage over medication (RR: −5.94; 95%; CI: −6.29 to −5.59; *P* < 0.00001; *I*^2^ = 99%; *n* = 252).

In previous reviews (Wang et al., 2008; Zhang et al., 2010, 2023; Armour et al., 2019; Chen et al., 2022), it was acknowledged that acupuncture, either alone or as an adjunct to pharmacological treatment, offers clinical benefits and can be considered as a safe therapeutic option for depression. However, many past reviews have had significant limitations concerning disease classification, inclusion criteria, and basis assessment. They often treat depression as a concomitant condition of a specific disease (Chan et al., 2015) rather than as a stand-alone condition. Furthermore, many studies included other interventions such as medication (Luo et al., 2021) or non-pharmacological treatments (Meng et al., 2021) in the control group, which has impacted the assessment of acupuncture’s effectiveness alone.

Acupuncture is recommended for treating mental disorders according to the National Guideline Clearinghouse (NGC),^1^ yet crucial factors such as point selection, stimulation modality, and duration of acupuncture are not specified (Guo et al., 2017). Multiple studies have confirmed that various types of antidepressant medication typically take 10 to 14 days to show onset of action, and it usually takes about a month to achieve full effect, with sustained improvement often requiring more than six months to ensure the stability of the medication’s efficacy and its effectiveness in neurological functioning (Yuan et al., 2020).

Of the 20 studies on acupuncture for depression included in this review, the vast majority employed a standardized acupuncture protocol, albeit with variations in frequency and treatment patterns between intervention and control groups. Our analyses indicate that acupuncture demonstrated comparable efficacy to medication during the initial 1–3 weeks. However, by the fourth week, acupuncture’s efficacy surpassed that of medication. This finding underscores the potential of acupuncture as a standalone therapy in depression treatment programs. For individualized patient needs, practitioners may consider acupuncture as a complementary approach to medication, particularly for patients who with slow or poor response to medication, strong adverse reactions, or those seeking non-pharmacological options. Acupuncture also serves as an alternative treatment option for patients hesitant about medication. The discovery that a treatment duration of at least 4 weeks is necessary can serve as a reference point for the onset of action in clinical treatment, aiding clinicians in refining treatment plans and assessing efficacy. While acupuncture has demonstrated significant efficacy in depression, its precise mechanism of action warrants further investigation. Future studies could explore how acupuncture influences neurobiological pathways and its interaction with pathological processes in depression. Additionally, research should continue to explore the effects of various acupuncture modalities, needle manipulation techniques, treatment frequency, and duration on depression treatment outcomes. In future RCTs, more comparisons between acupuncture and other non-pharmacological treatments, such as tuina, moxibustion, and psychotherapy, are warranted to evaluate the benefits of acupuncture in depression treatment.

### 4.2 Quality of the evidence

The RCTs analyzed in this study exhibited low risk for most items in the ROB assessment. They adhered to a strict 1:1 comparison ratio, featured larger sample sizes, stronger randomization and allocation concealment, higher confidence in outcome bias, and no data loss. However, only two studies (Dong and Xu, 2013; Gao and Tan, 2018) were considered to be at high risk for attrition bias. This was due to the lack of reporting reasons for case shedding in one study (Dong and Xu, 2013) and incomplete outcome reporting in another (Gao and Tan, 2018). Despite the comprehensive nature of acupuncture treatment, the mechanism of sham acupuncture remains incompletely understood (Liu et al., 2023). While comparative studies such as sham and placebo acupuncture have been conducted, the realization of double-blind interventions in RCTs has been challenging. Consequently, performance bias in the included studies was all evaluated as high risk.

### 4.3 Mechanism of acupuncture for depression

Acupuncture’s efficacy in treating depression may be associated with hippocampus, neurotransmitters, and JNK signaling pathway. The hippocampus, a crucial brain region for learning, memory, and emotion regulation, has been linked to depression, with studies showing a significant reduction in hippocampal volume in depressed patients (Dong et al., 2018), indicating that the hippocampus may have a direct correlation with depression. Needling depression model rats has been shown to significantly increase the levels of neurotransmitters such as 5-HT, NE, Ach, γ-GABA (Zhang et al., 2018), and Glu in the hippocampus, which play vital roles in emotion regulation, learning, and memory (Xiao et al., 2017). Furthermore, observations of the JNK signaling pathway in the prefrontal cortex of depressed rats revealed that acupuncture significantly decreased the levels of JNK protein, c-Jun amino-terminal kinase, and p-c-Jun protein (Yu et al., 2021). The JNK signaling pathway is crucial for the cellular stress response and is associated with neurological and cardiovascular diseases, suggesting that acupuncture’s mechanism of action in treating depression may involve its modulation of neuromonoamine transmitter content and activation of signaling pathways in brain tissues. In summary, these findings suggest that acupuncture’s efficacy in treating depression may be attributed to its modulation of neurotransmitters and signaling pathways in the brain. Although these studies provide valuable insights into acupuncture’s potential mechanisms for depression treatment, further research is needed to validate these findings and explore the specific mechanisms in more depth.

### 4.4 Acupuncture treatment regimen

Acupuncture point selection exhibited significant variability among the 20 included studies. Of the 20 studies included, a total of 43 points were mentioned, except for one study that did not specify the use of acupuncture points, where the choice of points varied considerably and none appeared independently. DU 20 was the most frequently utilized acupoint, with an application rate of exceeding 70%. Additionally, points such as EX-HN 3, LR 3, and PC 6 were commonly employed, each with an application rate exceeding 40%. Other potential points considered included HT 7, SP 6, ST 36, DU 24, EX-HN 1, and GB 20. Moreover, specialized points, such as temporal three and four needles, were occasionally selected. Interestingly, most of these points are not used in insolation. According to traditional Chinese medicine theory, acupoints serve as crucial connections between the body’s internal systems and the external environment. Considering the variability in disease manifestation among different patients, treatment plans must be highly individualized. TCM practitioners typically assess each patient’s specific condition to determine the combination of acupoints needed for stimulation to achieve therapeutic effects. As such, they are typically utilized in combinations rather than individually. This approach aligns with the holistic nature of acupuncture, which considers the clinical diversity of diseases at the time of onset. While there are variations in the selection of acupoints selection across studies, they all reflect the holistic approach of acupuncture to disease diagnosis.

Among those acupoints, DU 20 is regarded as a crucial therapeutic point, playing a pivotal role in raising yang and supplementing qi. Positioned at the apex of the head, it serves as the convergence point for yang qi from the three yang meridians of the hands, feet, and the vein of the head. Clinically, DU 20 is frequently utilized in treating disorders related to the head, brain, cardiovascular system, and neurogenic conditions such depression (Qu et al., 2023), anxiety (Li et al., 2022), stroke (Kim et al., 2011), hypertension (Zhou et al., 2022), and hemorrhoids (Feng, 2004). It is crucial to recognize that commonly used acupoints predominantly reside in the upper body and head region, which lack sufficient fat and muscle protection. Consequently, precise needle manipulation is imperative for practitioners. Furthermore, acupuncture sessions are typically lengthier lasting between 20 to 30 min, and necessitate continuous treatment for a minimum of four weeks.

## 5 Limitations

This study has several limitations that warrant consideration. Firstly, TCM treatment plans are highly personalized and difficult to replicate. Consequently, comparing results across studies becomes challenging due to heterogeneity in control group settings including variations in gender distribution, depression severity, and illness duration.

A second limitation concerns the low quality of many electroacupuncture studies in clinical settings, despite their large number. Specifically, the number of electroacupuncture studies meeting our strict selection and exclusion criteria was insufficient.

The third limitation arises from our exclusive focus on RCTs within the past decade, reflecting our prioritization of study currency and relevance. This approach, however, introduces a potential bias. Moreover, the variability in acupoint selection, specific acupuncture typologies, methodology, and the limitations of double-blinding present areas for improvement. Addressing these aspects could enhance the robustness of conclusions drawn from future research endeavors.

Fourth, all included studies originate from China, suggesting that acupuncture’s global use is not widespread. In the future, there is a need to enhance global awareness of acupuncture, train international acupuncturists, and conduct more cross-ethnic and cross-national research on RCTs.

## 6 Conclusion

This study conducted a comprehensive evaluation of the efficacy and safety of acupuncture as a stand-alone treatment for depression, employing rigorous study design and inclusion criteria. We meticulously controlled for sample comparisons between 1intervention and control groups, and rigorously selected RCTs that exclusively utilized acupuncture as a monotherapy. In this meta-analysis, 16 randomized controlled trials demonstrated that acupuncture treatment, administered for a minimum of four weeks, exhibited significant efficacy compared to pharmacological treatments. Particularly, acupuncture displayed fewer side effects and adverse reactions, suggesting potential benefits for depression patients over four weeks as opposed to medication alone. Further research is warranted to elucidate the specific acupuncture points and modalities most effective in treating depression.

## Data availability statement

The original contributions presented in this study are included in this article/supplementary materials, further inquiries can be directed to the corresponding authors.

## Authors contributions

YT: Writing – original draft, Writing – review & editing. RD: Writing – original draft, Writing – review & editing. CW: Writing – review & editing.

## Footnotes

1
http://www.guidelines.gov/
